# Synergistic Antifungal Study of PEGylated Graphene Oxides and Copper Nanoparticles against *Candida albicans*

**DOI:** 10.3390/nano10050819

**Published:** 2020-04-25

**Authors:** Yuen-Ki Cheong, Mariana P. Arce, Alejandro Benito, Daijie Chen, Noemi Luengo Crisóstomo, Laxmi V. Kerai, Guillermo Rodríguez, José L. Valverde, Mansukhlal Vadalia, Arisbel Cerpa-Naranjo, Guogang Ren

**Affiliations:** 1 School of Engineering and Computer Sciences, University of Hertfordshire, College Lane, Hatfield AL10 9AB, UK; y.cheong2@herts.ac.uk (Y.-K.C.);; 2 School of Architecture, Engineering and Design, European University of Madrid, C: Tajo s/n. Villaviciosa de Odón, 28670 Madrid, Spain; 3 School of Pharmacy, Shanghai Jiaotong University, 800 Dongchuan Road, Minhang District, Shanghai 200240, China; 4 School of Life and Medical Sciences, University of Hertfordshire, Hatfield AL10 9AB, UK; 5 R&D Department, Phi4Tech, Carretera de Ocaña 29, 45350 Noblejas, Spain

**Keywords:** antimicrobial nanoparticles, PEGylated graphene oxide, *Candida albicans*, copper nanoparticles (CuNPs), antifungal, minimum inhibitory concentration (MIC)

## Abstract

The coupling reactions of polyethylene glycol (PEG) with two different nano-carbonaceous materials, graphene oxide (GO) and expanded graphene oxide (EGO), were achieved by amide bond formations. These reactions yielded PEGylated graphene oxides, GO-PEG and EGO-PEG. Whilst presence of the newly formed amide links (NH-CO) were confirmed by FTIR stretches observed at 1732 cm^−1^ and 1712 cm^−1^, the associated Raman D- and G-bands resonated at 1311/1318 cm^−1^ and 1584/1595 cm^−1^ had shown the carbonaceous structures in both PEGylated products remain unchanged. Whilst SEM images revealed the nano-sheet structures in all the GO derivatives (GO/EGO and GO-PEG/EGO-PEG), TEM images clearly showed the nano-structures of both GO-PEG and EGO-PEG had undergone significant morphological changes from their starting materials after the PEGylated processes. The successful PEGylations were also indicated by the change of pH values measured in the starting GO/EGO (pH 2.6–3.3) and the PEGylated GO-PEG/EGO-PEG (pH 6.6–6.9) products. Initial antifungal activities of selective metallic nanomaterials (ZnO and Cu) and the four GO derivatives were screened against *Candida albicans* using the in vitro cut-well method. Whilst the haemocytometer count indicated GO-PEG and copper nanoparticles (CuNPs) exhibited the best antifungal effects, the corresponding SEM images showed *C. albicans* had, respectively, undergone extensive shrinkage and porosity deformations. Synergistic antifungal effects all GO derivatives in various ratio of CuNPs combinations were determined by assessing *C. albicans* viabilities using broth dilution assays. The best synergistic effects were observed when a 30:70 ratio of GO/GO-PEG combined with CuNPs, where MIC_50_ 185–225 μm/mL were recorded. Moreover, the decreased antifungal activities observed in EGO and EGO-PEG may be explained by their poor colloidal stability with increasing nanoparticle concentrations.

## 1. Introduction

Research in nanomaterials is rapidly growing, especially due to their potential for biomedical and healthcare applications. Graphene-based nanomaterials, such as carbon nanotubes and fullerenes, have created new avenues for researchers in nanomedicine, sensors, catalysis, etc. [1,2,3]. Their proven biocompatibility and low cytotoxicity have also attracted tremendous attention from biomedical scientists and pharmaceutical industries [4]. The most promising biological application involves using graphene as a delivery system for drugs; reports have shown that functionalised graphene can effectively shuttle small biological molecules into cells via the endocytic pathway, which is subsequently released at the target sites [5]. Moreover, their ability to form a vast number of functionalised graphene vehicles and to anticipate hydrophobic interactions in the central nervous system (CNS) have broadened the research interests in graphene chemistry and their potential in bio-medical engineering [6,7].

Graphene oxide (GO) is one of the functionalised graphitic derivatives, which has a well-known two-dimensional layer-structure that can be obtained by oxidative exfoliation of graphite [8]. Each fundamental layer of GO comprises of a sp^2^ hybridised carbonaceous skeleton forming a highly conjugated hexagonal structure, and each layer is held strongly by intermolecular covalent bonds [9]. Typically GO contains quaternary carbonyl groups, and it is also embedded with active epoxy- and carboxyl- groups that are situated at the structures exterior [10]. The presence of these functional groups facilitate surface modification via solution processing, including carboxylation and reduction. Due to the flexibility for surface functionalization of GO, the modified graphene oxides are ideal systems for studying the correlations between phase change behaviour and their surface properties. On the other hand, expanded graphene oxide (EGO) has a slightly different carbonaceous structure. EGO can be obtained by the reductions of graphite oxide [11] and is a promising precursor for high-quality products with enhanced properties due to its larger molecular surface area.

There have been numerous studies demonstrating antimicrobial properties of GO against a range of harmful pathogens, including both Gram-positive and -negative bacteria and biofilm forming microorganisms [12,13]. This antimicrobial effect is thought to be facilitated through both chemical and physical interactions of the graphene sheets with the bacterial cell membrane, which leads to morphological alterations within the cell [14]. It has been theorised that the sharp edges which form the flat atomic structure of graphene enable it to pierce through the cell membrane, thereby physically disrupting its integrity.

Conjugations of polyethylene glycol (PEG) with polymers is known to effectively improve biocompatibility [15]. Similarly, surface modifications of graphene using PEG (a process known as PEGylation), has also been widely investigated and promoted as a carrier to deliver hydrophobic anticancer drugs in mammalian systems [16]. PEG is a very useful biological reagent because of its minimal cytotoxicity, high biocompatibility and protein-resistant nature. It also has good solubility in water and is miscible in most common organic solvents for chemical synthesis [17,18,19,20]. PEG in the PEGylated GO has also been used as an inhibitor of bacterial wall synthesis [21,22], and antimicrobial synergistic effects have been reported when these PEGylated GO systems were combined with silver nanoparticles [23,24,25,26,27].

Candidiasis is one of the most frequent fungal infections in hospitals today [28], caused largely by the number of immunocompromised patients who are at a greater risk of developing this systemic fungal infection [29,30,31]. Of the *Candida* species responsible, *Candida albicans* (*C. albicans)* remains the most prevalent. One important factor that contributes to the pathogenesis of *C. albicans* is biofilm formation, as these fungal cells can form biofilms on biological and inert surfaces. Catheter-related infections are often caused by the *C. albicans’* ability to form biofilms, which can often be fatal, exacerbated by the growing resistance against most currently available antifungals agents [29,32]. Once the biofilm has formed, the sessile cells contained inside the biofilm have been proven to withstand some of the most effective drugs that are used to treat other yeast infections [33,34]. These are some of the major factors which attribute towards high mortality rates in patients with aggressive forms of candidiasis among various other clinical failures [35]. Due to the growing antifungal resistance of *C. albicans* along with the existing global concern of antimicrobial resistance (AMR) from other deadly pathogenic bacteria, research into alternative sources to combat AMR is becoming increasingly important.

**Figure 1:**
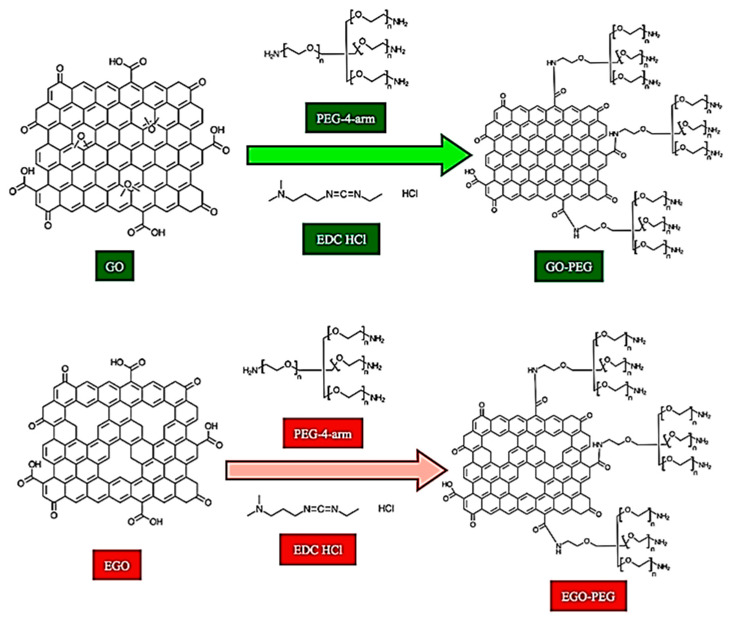
Synthesis of GO-PEG and EGO-PEG. As indicated in the image (UP) 4arm-PEG5K-NH_2_ was used as the PEG coupling reagent, while 1-ethyl-3-(3-dimethylaminopropyl) carbodiimide hypochlorite (EDC, HCl) was employed as the carboxyl activating regent to enable amide bond formation during the chemical conjugation.

In this study, we first report the synthesis of two different PEGylated graphene oxides (GO-PEG and EGO-PEG) that were prepared via amide bond formations as shown in Figure 1. In order to understand the structure activity relationship between these nano-graphene oxides and microorganisms, the physical and chemical characteristics of these PEGylated products were investigated using FTIR and Raman spectroscopy, and scanning and transmission electron microscopies (SEM and TEM). Furthermore, the physiochemical behaviour of these GO and PEGylated GO derivatives were studied by measuring their surface charges (and pH), hydrodynamic sizes and particle distributions using dynamic light scattering (DLS) techniques. Considering nano-silver has becoming a more common antimicrobial reagent and that microorganisms may become resistant to nano-silver in the near future, therefore, we chose to use copper nanoparticles (CuNPs) in our study and to investigate the potential synergistic antifungal activities in the presence of different graphene oxides. Antifungal activities of a total of five individual standard nanomaterials, including two graphene oxide standards (GO and EGO), their PEGylated derivatives (GO-PEG and EGO-PEG) and copper nanoparticles (CuNPs) were initially evaluated using an agar plate ‘cut-well’ method. The antifungal susceptibilities and synergistic effects were finally quantified by the minimum inhibitory concentrations (MIC) of each GO/CuNPs and GO-PEG/CuNPs nanocomposites using broth microdilution methods.

## 2. Materials and Methods

### 2.1. Nanomaterials

As shown in Figure 1, two different graphene oxides nano-sheets (GO and EGO) were employed and used as precursors of GO-PEG and EGO-PEG. Graphene oxide (GO) with a specific surface area of 20–35 m^2^/g and median mesoporous pore diameter of 127.5 Å was provided by NanoInnova Technologies SL. (Madrid, Spain). Expanded graphene oxide (EGO) with a specific surface area of 116 m^2^/g and median mesoporous pore diameter of 220 Å was provided by Castilla-La Mancha University (Madrid, Spain). EGO was prepared using a pilot method reported by Lee et al. [11]. Elemental copper nanoparticles (CuNPs) were spherical with a particle sizes ranging from 10–40 nm and was manufactured by CF Nanotechnology^®^ (Suzhou, China).

### 2.2. Synthesis of PEGylated Graphene Oxides (GO-PEG and EGO-PEG)

For the PEGylation of GO and EGO (Figure 1), 4arm-PEG5K-NH_2_ was used as the PEG coupling reagent, while 1-ethyl-3- (3-dimethylaminopropyl)carbodiimide hypochlorite (EDC HCl) was employed as the carboxyl activating regent to enable amide bond formation during the chemical conjugation. Both chemicals were purchased from Sigma-Aldrich (Madrid, Spain). All materials were used as received unless otherwise indicated. The GO-PEG and EGO-PEG were synthesised by modification of the method reported by Zhu et al. [21].

A mixture of graphene oxide nanoparticles (100 mg) and 4arm-PEG-NH_2_ (300 mg) were suspended in deionized water (100 mL) which was bath-sonicated for 5 min. EDC HCl (30 mg) was then added and the mixture was bath-sonicated for a further 40 min at room temperature, this was followed by further addition of EDC HCl (80 mg) and the mixture was stirred overnight. The resulting crude reaction mixtures were subjected to centrifugation (Digicen 21 centrifuge, Orto Alresa Spain) at 5000 rpm to remove any unreacted PEG. The supernatant was then removed and the solid was washed and subsequently filtered under vacuum. Finally, the PEGylated product was filtered to dryness under reduced pressure and then air dried for 24 h at room temperature. The same experimental procedure was employed to obtain EGO-PEG. Both GO-PEG and EGO-PEG were isolated as black solids.

### 2.3. Nanoparticle Dispersions for Physiochemical and Antifungal Testing

In this study, GO, EGO, GO-PEG, EGO-PEG and CuNPs dispersions with varying ratios were prepared for physiochemical and antifungal evaluations. A 0.1% *w*/*v* stock suspension of each nanoparticle dispersion was prepared by first suspending 20 mg of nanocomposites into 20 mL of pure water (Acros, Organics-Fisher scientific UK), which was then radiated in a Vibra-cell 750 ultrasonic liquid processor (Sonics and Materials^®^, Newtown, CT, USA) for 2–3 min at 50% working amplitude with an applied pulse sequence (20 s on and 5 s off). All suspensions were cooled either in ice/water bath immediately after each dispersion process. When required, different elemental ratios of nanocomposites were prepared freshly using standard dilution method.

### 2.4. FT-IR Spectroscopy

Infrared spectra of GO, GO-PEG, EGO and EGO-PEG samples were measured in a FT-IR Nicolet iS50 (Thermo Scientific, Spain) instrument, equipped with ATR germanium technique (Miracle™ Single Reflection ATR, Pike, Spain). Powder samples were loaded directly onto the germanium crystal stage and secured by a compressor rod. Blanks were performed prior to each sample submission; all spectra were measured at a wavenumber range between 3500–500 cm^−1^.

### 2.5. Raman Spectroscopy

Raman spectra were obtained by a Renishaw inVia Raman microscope (Gloucestershire, UK) equipped with a WiRE 3.4 software supplied by the manufacturer. All measurements were performed at 785 nm excitation wavelength and a 2 mW power laser. All powder samples were presented on a microscope slide with an approximate examined area of 20 × 20 μm^2^ and each measurement was taken after an average of 20 scans. The data were further processed using the BioRed^®^ (Philadelphia, PA, USA) program and all visible Raman shifts were studied against the references supported by the database within the program.

### 2.6. SEM and TEM Microscopy

The surface morphologies of GO, GO-PEG, EGO and EGO-PEG nanoparticles were studied using scanning electron microscopy (SEM) and transmission electron microscopy (TEM). All the SEM images for GO, EGO and their PEG-derivatives were acquired using an X-Max Oxford Instrument (Oxford, UK) at an operating power range between 127 eV and 5.9 kV. One drop of diluted suspension of each nanoparticle sample was placed on a silicon wafer, which was attached to an aluminium sample stub with a conductive carbon adhesive. All the biological samples were fixed using 2.5% glutaraldehyde and 2% osmium tetroxide, washed with phosphate butter and gradient mixtures of ethanol/water [36]. Yeast samples were then dehydrated with acetone and air dried for 24 h. All biological samples were mounted on SEM stubs the same way as described before, and all SEM images of biological samples were acquired using a JEOL JCM-5700 (Welwyn Garden City, UK) instrument. All SEM samples were dried under vacuum and coated with 2–3 nm of gold using an Emitech SC7620 sputter (Quorum Technologies, Ltd., East Sussex, UK) prior imaging acquisitions. All images were collected and processed using in-built software within the instrument. TEM images of GO and EGO samples were acquired using a JEOL 2100 instrument (Tokyo, Japan) operating at 200 kV. For this study, a few drops of each dispersions containing a concentration of 0.005 mg/mL of solids were placed on a copper grid and volatile solvents were evaporated prior to observation.

### 2.7. Colloidal Stability and pH Measurement of Nanoparticle Dispersions

Zeta-potentials of GO, GO-PEG, EGO and EGO-PEG aqueous dispersants at concentrations of 10, 50 and 100 ppm were measured using the micro-electrophoresis technique equipped with a laser Doppler velocimetry (Zetasizer Nano ZS, Malvern, UK). All pH values were measured at seven different concentrations (10, 25, 50, 100, 250, 500 and 1000 ppm) using an Accumet™ AP110 Portable pH Meter (Fisherbrand™, Leicestershire, UK). The pH probe was calibrated using pH 7 and pH 4 buffer between analysis of different samples. All zeta potential and pH values of dispersants were measured at 22.0 ± 0.1 °C. All values reported were determined from the mean values of at least three successive measurements.

### 2.8. Nanoparticle Tracking Analysis at Real-Time

Nanoparticle tracking analyses were performed using a NanoSight LM14 (Malvern Instruments, UK) equipped with a green laser source (532 nm) and a CMOS camera. Each nanoparticles dispersant of 0.005% *w*/*v* (50 ppm), 0.01% *w*/*v* (100 ppm) and 0.025% *w*/*v* (250 ppm) was directly loaded into sample chamber using a syringe, detection was measured under a static mode at room temperature. Real-time visualisation of the nanoparticles was optimised by adjusting the NTA camera shutter, gain and laser power. All nanoparticles in each sample were tracked and captured for 30 s in a single analysis, which were acquired in three technical and software replicates. Hydrodynamic size ranges and particle concentrations were obtained from means values of at least three successful replicates. All data was collected using in-built software (NTA 3.2 Dev Builds 3.2.16) and was further processed using MS Excel (Microsoft, Redmond, WA, USA).

### 2.9. Cultures and Biological Reagents

*Candida albicans* (ACTT 2091) was obtained from the University of Hertfordshire collection (Life and Medical Sciences), Mueller-Hinton (MH) agar, Sabouraud Dextrose Agar (SDA), yeast peptone dextrose agar (YPD) along with other agar/broth ingredients were purchased from Sigma-Aldrich, UK. All the saline and buffer solution used in this study were freshly prepared from the bulk powder (SIGMA-Aldrich, UK) according to manufacture guidance and were all sterilised in an autoclave (121 °C, 15–20 min) prior to in vitro studies. Trypan Blue (Sigma-Aldrich, UK) was used to stain dead cells while counting viable colonies using a haemocytometer. Other bio-reagents for performing MIC assays, which included RPMI 1640 L-glutamine with sodium bicarbonate, Clotrimazole, PBS, XTT sodium salt and Menadione, were all purchased at Sigma-Aldrich (Dorset, UK). The materials were stored at room temperature, except RPMI 1640 medium which was stored at −20 °C until used.

### 2.10. Susceptibility Tests of Standard Alone Nanomaterials against Candida Albicans

A modification of the ‘cut-well’ method [37] was used to screen antifungal activities of standard nanomaterials (ZnO, Cu, GO, EGO, GO-PEG and EGO-PEG) against *C. albicans* (*ATCC 2091*) as the model organism. *C. albicans* was aerobically cultivated in SDA for 48 h at 30 °C. Colonies were taken with a sterile loop and suspended in 7 mL of sterile Ringer saline. The mixture was then vortexed for 15 s and the cell population was adjusted to 1 × 10^6^ to 1 × 10^8^ colony-forming units (cfu)/mL. The yeast suspension (150 µL) was inoculated on previously ‘well-cut’ YPD agar plates using a sterile glass spreader. Negative controls (pure water) and three replicates of different standard nanomaterials dispersants (18 µL) at a concentration of 0.1%w/v (1000 ppm) were pipetted into each well (5 mm × 6 mm, diameter × depth) on the yeast inoculated agar plates, all treated yeast cultures were then incubated at 30 °C for 48 h. After the incubation period, inhibitory effect could be observed by the absence of visible colonies. To quantify colonies in each treated yeast well, fresh Ringer saline (18 µL) was added into each well, which was then transferred into an Eppendorf and further diluted with mixture of Ringer saline and Trypan blue (0.025%). Quantification of the treated and controlled yeast cells were performed using a haemocytometer. A susceptibility test was repeated twice, a total of six replicates of viable yeast-counts were obtained and the raw data was processed using MS Excel (Microsoft, Redmond, WA, USA).

### 2.11. Determination of Minimum Inhibitory Concentrations (MIC) of Nanomaterial Composites

The in vitro synergistic antifungal study was performed using four different ratios of graphene oxide derivatives (GO, EGO, GO-PEG and EGO-PEG) with CuNPs. The GO:Cu ratios were 96:4, 70:30, 30:70 and 1:99, respectively. MIC assays of a total of 16 combinations were achieved using a modification method that was outlined by Pierce et al. [38].

*C. albicans* (ATCC 2091) was cultured overnight in YPD agar for 24 h at 30 °C. A colony of the fresh *C. albicans* was suspended into 1 mL of phosphate-buffered saline (PBS) and the mixture was centrifuged (Hettich centrifuge EBA200, Bäch, Switzerland) at 6000 RPM for 5 min. The resulting *C. albicans* cells were washed again with PBS and then re-suspended in 1 mL of RPMI 1640 medium. Cell forming unit (cfu/mL) of *C. albicans* was measured using a haemocytometer and the concentration of the cells was re-adjusted to 1.0 × 10^6^ cells/mL in RPMI 1640 medium. A total of 100 µL of the *C. albicans* broth and 100 µL of the corresponding the Graphene Oxide and Copper nanoparticle ratio (96:4, 70:30, 30:70 and 1:99) at different concentrations (0, 7, 15, 31, 62, 125, 250, 500 µg/mL) were then added into a 96 multiwell plate, and the mixtures were then incubated at 30 °C for 24 h.

A saturated XTT reagent was prepared by dissolving 0.5 g of XTT powder into 1 L of PBS and this solution was covered in foil during preparation. A10 mL working volume of the XTT reagent was filtered using MilliPore syringe filters (0.22 μm-pore size) and stored at −70 °C before used. For the Menadione reagent, a 10 mM stock solution in 100% acetone was prepared and stored at −70 °C before used. After 24 h of incubating the *C. albicans* cells in nanoparticle suspensions, 100 µL of the freshly prepared XTT/Menadione reagent (1 μL Menadione + 10 mL XTT) was added to each well of the 96 multi-well plate. The plate was covered in foil and incubated for further two hours at 37 °C before O.D. values (λ 490 nm) was obtained from using a micro-plate colorimeter (CLARIOstar BMG Labtech, Offenburg, Germany). All the viability values presented were obtained from average values of replicates of two successful experiments. The MIC_50_ values represent the lowest antifungal concentration leading to >50% reduction in colorimetric readings.

## 3. Results and Discussion

### 3.1. Chemical and Morphological Characterization of GO, EGO, GO-PEG and EGO-PEG

**Figure 2:**
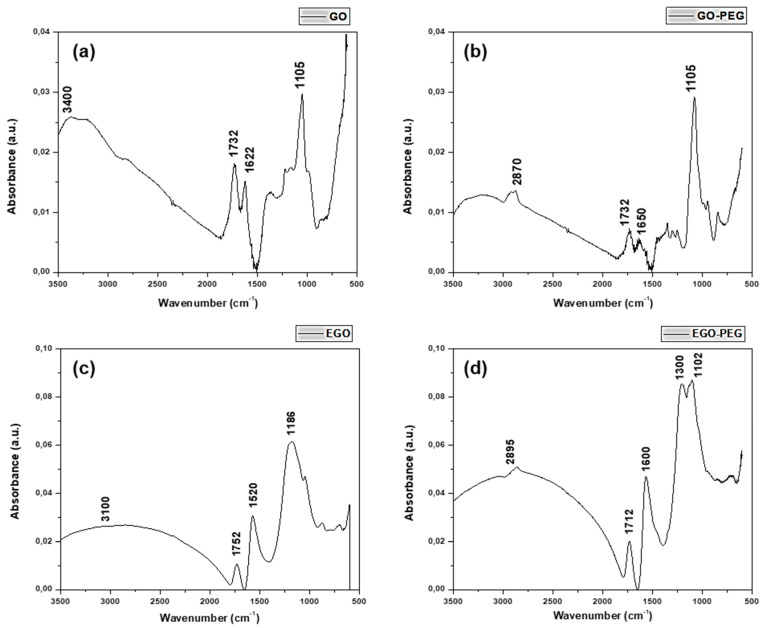
FTIR spectra of (**a**) GO, (**b**) GO-PEG, (**c**) EGO and (**d**) EGO-PEG.

Figure 2a–d shows the FTIR spectra obtained from the four different graphene oxide derivatives, GO, GO-PEG, EGO, and EGO-PEG. The FTIR spectrum of GO (Figure 2a) shows two vibrational bands at 1105 and 1732 cm^−1^, corresponding to the C–OH and C=O stretches from the carboxylic acid group (–COOH) within the graphitic structure. Whereas the band resonated at 1622 cm^−1^ was attributed to the bending mode in the sp^2^ hybridised C=C within the highly conjugated GO structure. The broad signal observed between 3500–3000 cm^−1^ can be attributed to OH stretching due to intermolecular hydrogen bond interactions between the carboxylic acid groups (COOH) presence on the outskirt of the GO structure. Thus, the IR spectrum of GO is consistent with that reported by Marcano et al. [39].

In contrast, a new band was found in the FTIR spectrum of GO-PEG (Figure 2b) at 2870 cm^−1^ and this corresponded to the stretching of the C–H bonds derived from PEG coupling. As expected, an amide signal (NH–CO) was observed at 1732 cm^−1^ after the amidation coupling reaction during PEGylation. Although resonated at the same wavelength with GO, however, the C=O signal in the PEGylated product was much weaker [40].

Figure 2c,d show the FTIR spectra of EGO and EGO-PEG respectively. The very broad bands at 3250–2250 cm^−1^ appeared in both EGO (Figure 2c) and EGO-PEG (Figure 2d) suggested that the samples were hygroscopic. The increased hygroscopicity found in the resulting EGO-PEG made it difficult to identify the N-H stretch, which was expected after the PEGylation process. Although the FTIR spectra of EGO (Figure 2c) and EGO-PEG (Figure 2d) were not as resolved, the C=O stretches observed in the corresponding acidic EGO were quite different to the PEGlyated product (1752 cm^−1^ vs. 1712 cm^−1^). The band observed at 1712 cm^−1^ EGO-PEG (Figure 2d) was therefore associated with the amide group (NH–CO) formed after the EGO had chemically coupled with PEG as shown in Figure 1. In contrast, the band at 1752 cm^−1^ observed in the EGO sample, would be a characteristic carbonyl (C=O) stretch associated to the carboxylic acid group in the starting material.

The above evidence suggested that the coupling reaction of PEG with the two types of graphene oxides (GO and EGO) had successfully occurred and that new amide bonds were formed yielding PEGylated graphene oxide (GO-PEG) and PEGylated expanded graphene oxide (EGO-PEG).

**Figure 3:**
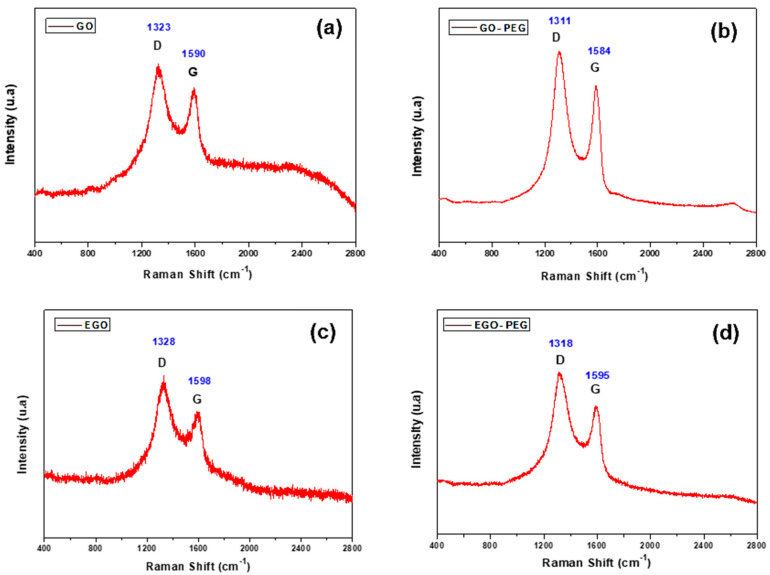
Raman spectra of (**a**) GO, (**b**) GO-PEG, (**c**) EGO and (**d**) EGO-PEG.

In addition to the FTIR study, the four different graphene oxide derivatives were also analysed using complementary Raman spectroscopy. Figure 3a–d show the Raman spectra of GO, GO-PEG, EGO and EGO-PEG, respectively. Two distinctive signals, the D and G bands were observed within the range of 1300–1650 cm^−1^ in all four samples, which were attributed to the presence of sp^3^/sp^2^ hybridized carbon-carbon bonds in the graphitic compounds. Unlike the pristine graphene, the strong and broad D band and high I(D)/I(G) ratio in our GO samples indicated their lattice distortions and large amount of sp^3^-like defects due to their oxidised nature [41]. This Raman feature is quite commonly found in graphene oxide species.

Figure 3a shows the Raman spectrum of GO which shows peak D at 1323 cm^−1^ and peak G at 1590 cm^−1^, while in the EGO sample peak D appeared at 1328 cm^−1^ and peak G at 1598 cm^−1^, confirming the distortions of the network (Figure 3a,c). In the case of the GO-PEG conjugated materials (Figure 3b) and EGO-PEG (Figure 3d), the D and G bands were shifted to give lower wavenumbers at 1311 cm^−1^ and 1318 cm^−1^ for the D bands and 1584 cm^−1^ and 1595 cm^−1^ for G bands, respectively. In the case of band D, the displacement was more pronounced. It was also observed that the signal to noise ratios were much better resolved in the PEGylated samples when comparing to the starting GO/EGO, hence, the addition of the organic moiety (i.e., the PEG groups) had enhanced light scattering intensities.

**Figure 4:**
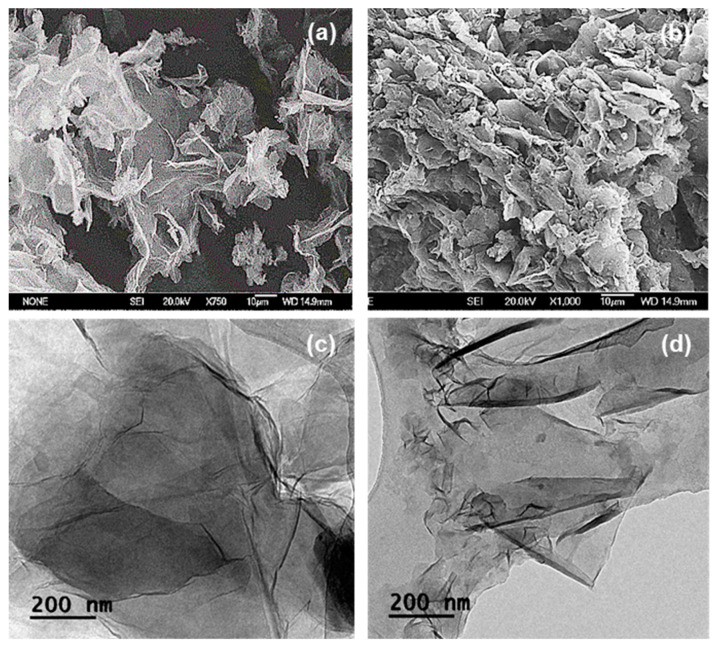
SEM of (**a**) GO and (**b**) GO-PEG. TEM of (**c**) GO and (**d**) GO-PEG.

Figure 4a,b show the SEM images of GO and GO-PEG. As shown in Figure 4a, GO demonstrated a sheet-liked structure with sharp edges and a smooth surface. Although the subsequent PEGylated product, GO-PEG appeared to be heavily agglomerated, a laminar nano-layer structure can still be seen in the corresponding SEM image (Figure 4b). The observed agglomerations and a more randomised structure in GO-PEG can be explained by the spatial interactions between the newly formed PEG-arms, of which had grafted onto the edges of the GO layers [42]. Subsequently giving rise to the thickness increase, similar effect has been reported by Lu et al. upon the functionalisation with aminoethanesulfonic acid via amide bond formations [43].

Figure 4c,d shows the TEM micrographs of GO and GO-PEG, as illustrations in dispersed and singular forms. The TEM image of GO (Figure 4c) depicted very thin and transparent sheets, whereas the corresponding PEGylated product, GO-PEG appeared to have deformed edges. Again, such morphological changes were expected, due to the presence of additional PEG groups situated on the surface of the GO layer, hence, the layer had become denser.

**Figure 5:**
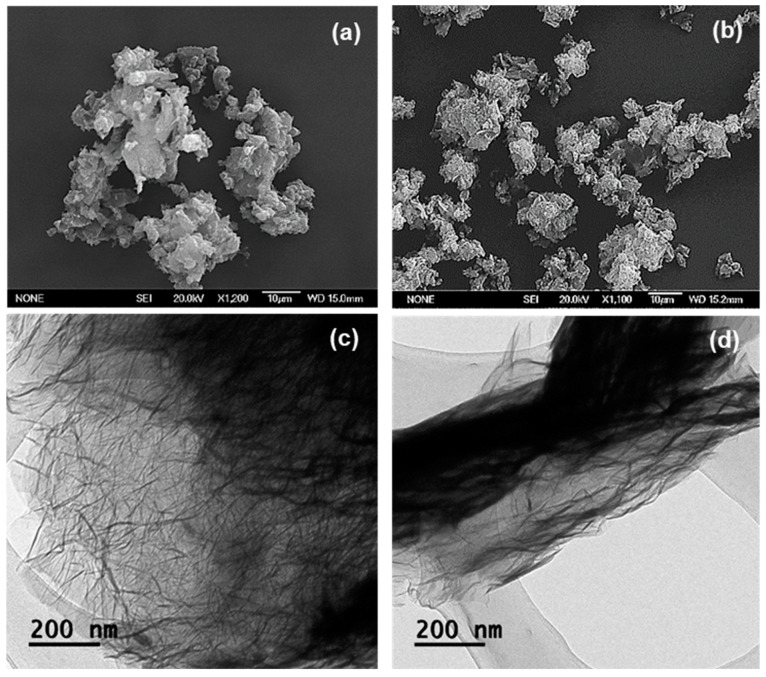
SEM of (**a**) EGO and (**b**) EGO-PEG. TEM of (**c**) EGO and (**d**) EGO-PEG.

Figure 5a,b shows SEM micrographs obtained from EGO and EGO-PEG. As can be seen in Figure 5a, these expanded graphene oxide (EGO) derivatives still appeared as a layered-structure, but heavily agglomerated. The particle surface areas in EGO and EGO-PEG were found to be much smaller than those were seen in the GO and GO-PEG nano-sheets (Figure 4a,b). This physical behaviour was also observed in the corresponding TEM image.

Unlike the discrete single layer structure observed in GO (Figure 4c), EGO appeared to have a more fluff-liked and branched-out feature (Figure 5c) [43]. The layers in EGO were weakly stacked onto each other to form the apparent depths that can be seen in the darker area. On the other hand, the PEGylated expanded graphene oxide (EGO-PEG), obtained from the coupling reaction of EGO, had shown to have reduced in particle sizes, this can be seen in the corresponding SEM image (Figure 5b). Similar to the GO-PEG (Figure 4d), darker particle layers and greater depth density were observed in the EGO-PEG TEM image (Figure 5d) due to the additional PEG arms situated on the outer layer of EGO. Interestingly, the PEGylated EGO-PEG appeared to have lost the branch-out feature and, instead, it was re-organised to give a more aligned fibrous-like structure with the apparent increased depths that we reported in the GO-PEG (Figure 4d). These observations suggested that introduction of covalent linkages to EGO had not only changed the intra-molecular structure to give EGO-PEG, but also changed the inter-molecular behaviour in these macromolecules.

In summary, newly formed amide bonds were identified in the FTIR spectra (Figure 2a–d) after the PEG coupling reaction of both GO and EGO, which suggested that the PEG groups were successfully coupled to the two types of graphene oxides using the synthetic method reported by Zhu et al. [21]. (Figure 1). The consistent resonances observed in Raman spectra for the four graphene oxide derivatives (Figure 3a–d) indicated that the carbonaceous structures of the PEGylated products (GO-PEG and EGO-PEG) remained unchanged after the coupling reactions. It is interesting to observe that the resolutions of the Raman signals in both PEGylated products were improved after additions of the hydrophilic groups to GO and EGO. SEM imaging of all four graphene oxide derivatives (Figure 4a,b and Figure 5a,b) revealed the nature of these nano-powders in bulk, while the TEM images (Figure 4c,d and Figure 5c,d) illustrated their true forms following dispersion in an aqueous medium. The former also represented the physical relationships of these graphene oxide nano-sheets with the physiochemical and antifungal evaluations that we report herein.

### 3.2. Physiochemical Characterisations of GO, GO-PEG, EGO and EGO-PEG

**Figure 6:**
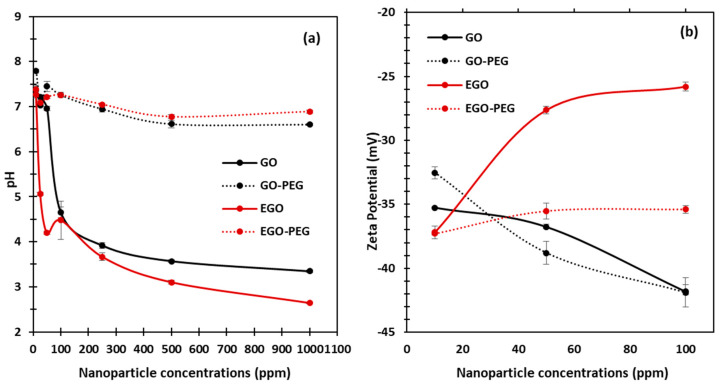
(**a**) pH measurement of GO, GO-PEG, EGO and EGO-PEG dispersants at seven different concentrations (10, 25, 50, 100, 250, 500 and 1000 ppm), with pH of blank water measured at 7.22 (+0.09). (**b**) Zeta potentials of GO, GO-PEG, EGO and EGO-PEG dispersants were measured at three different concentrations (10, 50 and 100 ppm).

With an aim to understand how different graphene oxide derivatives may have interacted and attributed to the observed antifungal effects against *C. albicans*, we investigated the physiochemical behavior of each standard alone, i.e., GO, GO-PEG, EGO, and EGO-PEG, at different aqueous concentrations. As shown in Figure 6a, the pH levels of each graphene oxide derivative were measured at seven different dilutions (10, 25, 50, 100, 250, 500 and 1000 ppm). The pH observed clearly shows that both starting GO and EGO were acidic, as their concentrations increased from 10 ppm to 1000 ppm, the pH values of both GO and EGO dropped significantly to 3.3 (± 0.03) and 2.6 (± 0.02), respectively. It is worth noting that the pH levels of both GO-PEG and EGO-PEG stabilised to near neutral pH level (~pH 7) at all concentrations. This provided evidence that successful PEGylation had occurred during the coupling syntheses. In order to determine the surface charges and to understand the colloidal stabilities of the graphene oxide nanoparticle derivatives, zeta potentials of GO, GO-PEG, EGO and EGO-PEG dispersants were measured at concentrations 10, 50 and 100 ppm (Figure 6b).

As indicated in Figure 6b, all standard alone graphene oxide derivatives had negatively-charged particle surfaces between −25.8 to −41.9 mV. Interestingly, both GO and GO-PEG showed increased hydrodynamic stabilities as their negative zeta values were larger at higher concentrations (i.e., 41.9 mV at 100 ppm). Good particle stability is expected to increase the exposure time during physical interactions between nanoparticles and microorganisms, which may enhance antifungal effect. In contrast, both EGO and EGO-PEG particles showed be less stable in their aqueous states as their concentrations increased, this can be seen from the small negative zeta values obtained at 100 ppm (25.8 and 35.4 mV).

**Figure 7:**
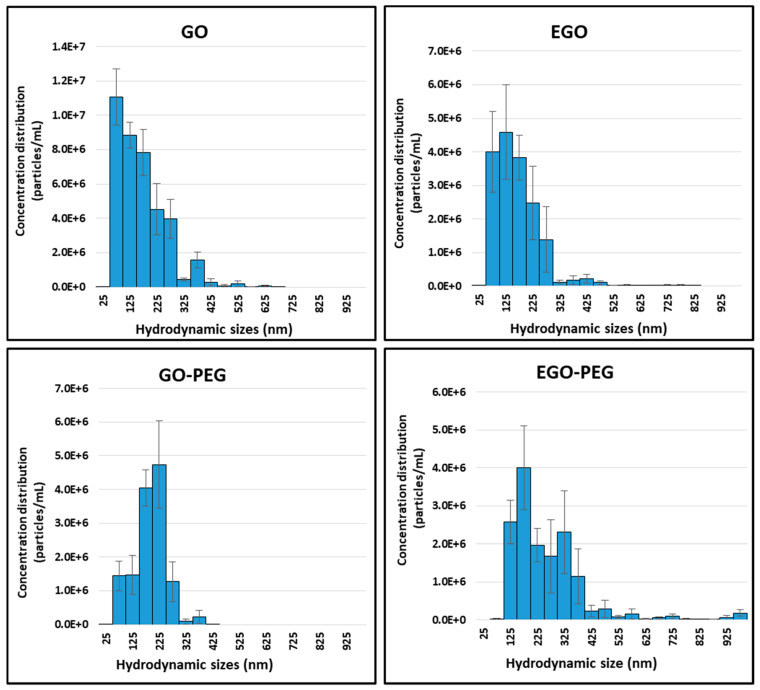
Nanoparticle tracking analysis of GO, EGO, GO-PEG and EGO-PEG showing the particle sizes in relations to their concentrations and distributions in aqueous dispersants.

As expected, the particle sizes of these graphene oxide derivatives are fairly polydispersed, especially in their aqueous suspension forms. Nanoparticle tracking analyses were performed to investigate the hydrodynamic behaviour of the four standard alone GO, GO-PEG, EGO and EGO-PEG using dynamic light scattering (DLS) technique. The particle concentrations and distributions were measured by capturing individual nanoparticles at real-time. Figure 7 shows the particle concentrations, sizes and distributions in each graphene oxide sample. Of the samples, GO-PEG was found to have the most uniform hydrodynamic size range between 75–375 nm when compared to other three samples. In contrast, EGO-PEG appeared to have the most polydispersed property of all, where its hydrodynamic size range distributed across 125–425 nm and beyond.

### 3.3. Antimicrobial Activities of GO, GO-PEG, EGO and EGO-PEG against C. Albicans

**Figure 8:**
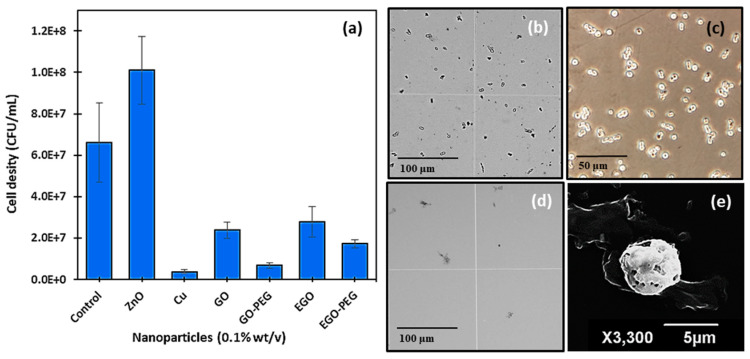
(**a**) Antifungal susceptibility test of different nanomaterials. Haemocytometer images of *C. albicans* control at (**b**) 10× magnification; (**c**) 60× magnification and (**d**) debris after treatment with CuNPs. (**e**) SEM image of affected *C. albicans* cells after exposure to CuNPs.

Initial antifungal activities of nanomaterial standard dispersants (0.1% *w*/*v*), copper nanoparticles (CuNPs), zinc oxide nanoparticles (ZnONPs) and the four graphene oxide derivatives (GO, GO-PEG, EGO and EGO-PEG) were tested against *C. albicans* using ‘cut-well’ method [37]. The ‘cut well’ method was repeated twice on separate agar plates and the antifungal activities of each nanomaterials were observed in triplicates. This method provided fast antifungal screening results from testing a large number of nanomaterial samples, it also allowed the semi-quantification of the growth or reduction of cell densities after nanoparticle exposures. In this study, ZnO was chosen as a negative control, as zinc is known to be a growth nutrient for yeast cells [44,45]. As expected, *C. albicans* counts (cfu/mL) increased to 1.0 × 10^8^ after exposure to ZnONPs (Figure 8a). In this preliminary antifungal study, standard alone CuNPs showed to be more efficacious against *C. albicans* when compared to all other graphene oxide derivatives, as the resulting cell density was low, and the inhibitory effect was clearly visualised through the haemocytometer (Figure 8d) when comparing with the control culture (Figure 8b,c). The lack of viable cells after CuNPs treatment had caused great difficulty when attempted to stain viable cells for further morphological studies using an optical microscope. After a few attempts, it was possible to locate affected yeast cells after treatment with CuNPs using SEM, the micrograph for which is shown in Figure 8e. As can be seen in Figure 8e, the yeast cell appeared to have undergone the initial state of apoptosis, where pores were formed on the surface of the cell. It was unfortunate that the different stages of apoptosis could not be captured, as further microscopic studies suggested that the anti-fungal effect observed in CuNPs occurred within 15–20 min.

**Figure 9:**
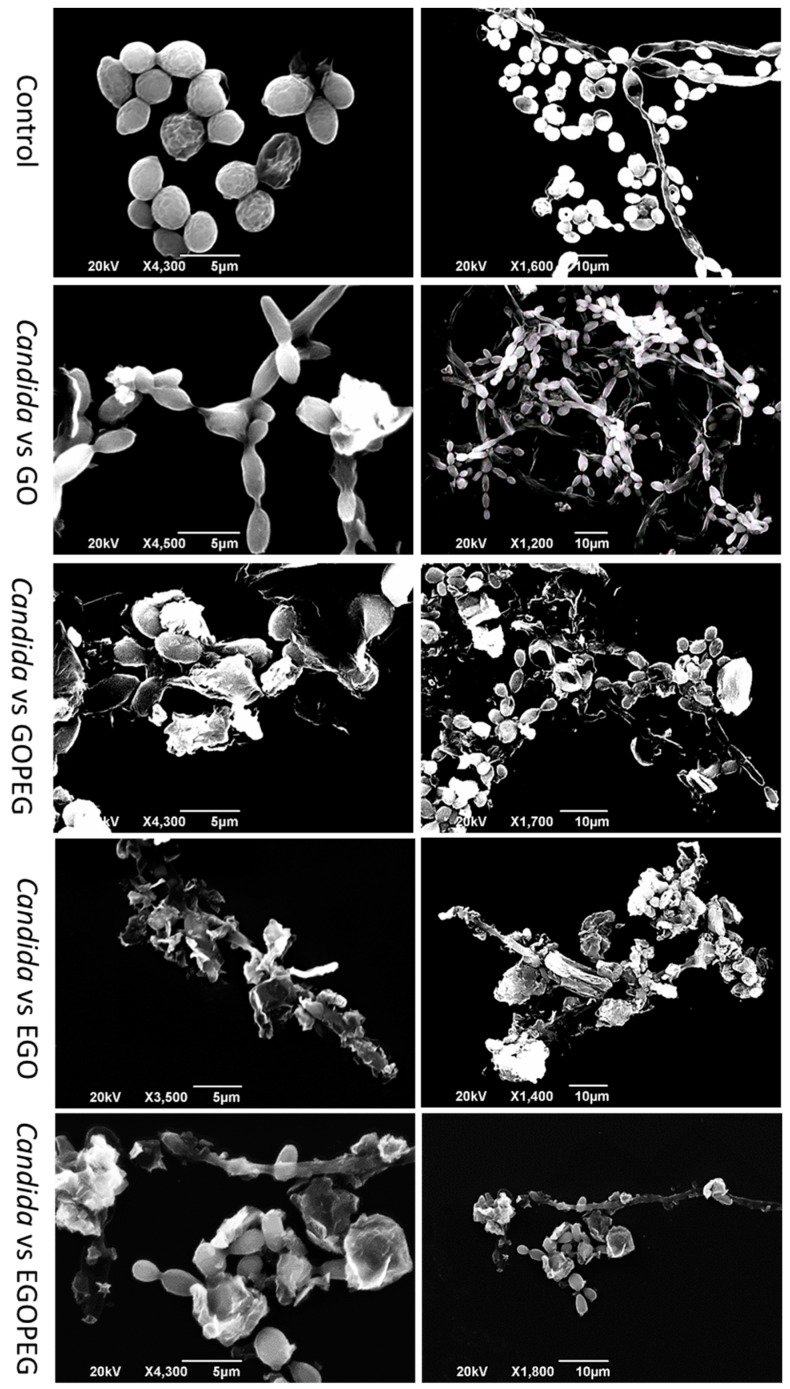
SEM images of *C. albicans* before and after treatments of standard alone GO, GO-PEG, EGO, and EGO-PEG nanomaterials.

With regards to the four graphene oxide derivatives, GO-PEG showed to be the best anti-fungal agent of all (Figure 8a). A mean cell counts of 3.7 × 10^6^ cfu/mL was measured; this was equivalent to approximately a three times reduction when compared to the control count (6.6 × 10^7^ cfu/mL). The reduction effect exhibited by the GO-PEG treatment was also observed in our later MIC study. As the anti-fungal effects observed from the different graphene oxides and their PEGylated derivatives were much slower than those of CuNPs. It was possible to capture the morphological changes of the affected yeast cells after 16 h of treatments. Figure 9 shows SEM images (at 5 μm and 10 μm magnifications) of the control *C. albicans* along with treated cells that were exposed to GO, GO-PEG, EGO and EGO-PEG, respectively.

In general, all *C. albicans* cultures that had been treated with graphene oxides underwent morphological changes. As shown in Figure 9 (top images), healthy yeast cells were grown which appeared in spherical shapes with diameters between 3–4 μm, and the bud to hyphae transition (filament) was observed in healthy cells as well as all treated cells. After treatment with GO, although the overall yeast structure maintained, the spores had clearly shrunk and changed from spherical to oval shapes. In comparison, the morphological changes in yeast spores for the GO-PEG treated cells, was more apparent, as clear debris remained from dead spores and hyphae which were still attached to the main branches. It is also worth noting that un-washed GO-PEG nano-sheets were also present and appeared to have tightly adhered to the yeast spores.

In comparison, after treatment with EGO, both yeast spores and hyphae suffered from heavy morphological deformation. While the filament structure was still present, the spores were mostly destroyed by the presence of EGO nano-sheets, which again can be seen in the SEM images (Figure 9). The exposure of EGO-PEG over the yeast cells did not seem to have caused as much damage as the corresponding precursor EGO, as SEM images showed both hyphae and spores, which although deformed, where still present.

### 3.4. Determination of a Synergistic Effect of GO, EGO and Their PEGylated Derivatives in the Presence of CuNPs

**Figure 10:**
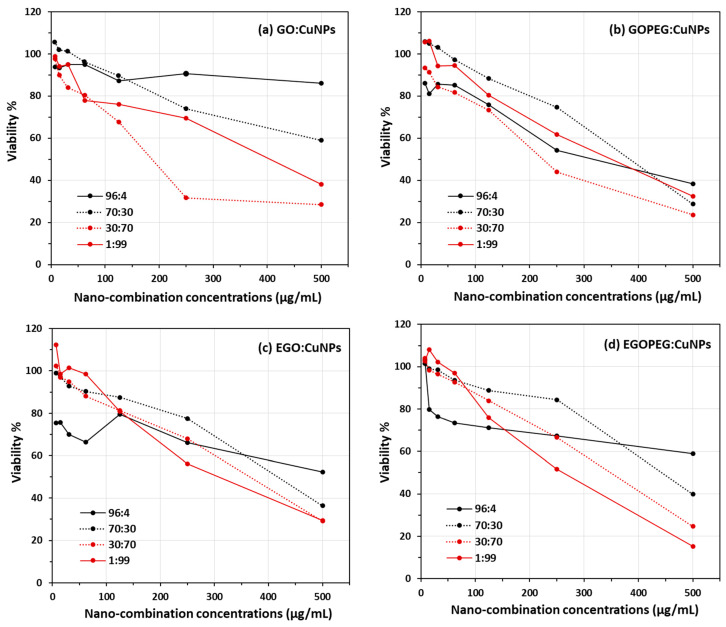
*C. albicans* viability assays obtained from combinations of (**a**) GO:CuNPs, (**b**) GO-PEG:CuNPs, (**c**) EGO:CuNPs and (**d**) EGO-PEG:CuNPs. Four GO:CuNPs ratios were studied, including 96:4, 30:70, 70:30 and 1:99.

As mentioned in the previous section, the initial cell count studies using the cut-well method provided an insight of the antifungal effect of different nanomaterials. However, it was difficult to justify the accuracy of each count, as dead cells often appeared as debris and still stained following treatment with Trypan Blue. Therefore, it was impossible to unambiguously identify viable yeast cells appropriately using the haemocytometer. In order to quantify viable yeast cells and to evaluate the synergistic antifungal effects of these graphene oxide derivatives, MIC assays were performed by adding different ratios of copper nanoparticles (CuNPs) to GO, GO-PEG, EGO or EGO-PEG (Figure 10 and Table 1). The viability curves (Figure 10) were obtained from reading the absorbance at λ 490 nm produced by viable cells after quenching its reaction with the nanomaterials combinations using XTT. Clear trends of reductions can be seen as concentrations of nanoparticles increased from all 16 experiments (Figure 10a–d).

Figure 10a–d show the viability curves of *C. albicans* after treatments of different GO:CuNPs combinations using broth dilution method (500, 250, 125, 62, 31, 15 and 7 μm/mL). Four different GO:CuNPs ratio combinations included 96:4, 70:30, 30:70 and 1:99 respectively were chosen in this study. Instead of testing the standard alone GO derivatives and the CuNPs, we decided to manipulate the respective GO:CuNPs ratios to 96:4 (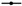) and 1:99 (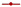), in hope to see any obvious synergistic indication. In the presence of 4% CuNPs, GO-PEG showed to exhibit much better antifungal activities when compared to its precursor GO (Figure 10a,b). This can also be seen in Table 1, at concentrations 500 μm/mL, the *Candida* viability decreased from 86% to 38% after exposure to GO and GO-PEG with the minimal added CuNPs. Similarly, at concentrations 250 μm/mL, *Candida* viabilities also decreased from 91% to 54% respective to the exposures with GO/GOPEG with 4% of CuNPs. This results also agreed with the initial antifungal screening using the agar ‘cut-well’ method (Figure 8a), where lower cell density was encountered in the GO-PEG standard alone treatment. The observed antifungal ability in GO-PEG may be explained by the high colloidal values obtained from the zeta potential measurements (−41.9 mV) and the mono-dispersive behaviour observed in the NTA study (Figure 7).

The best MIC_50_ values were found when 30:70 combinations were employed in both GO:CuNPs (185 μm/mL) and GOPEG:CuNPs (225 μm/mL). Although the 30:70 GO:CuNPs combination appeared to have lower inhibitory concentration for deactivating half (MIC_50_) of the yeast colonies, the corresponding GOPEG:CuNPs appeared to have the deactivations more directly proportional to the nanoparticle concentrations. Thus resulting a slightly lower viability values at higher concentration (Table 1), for instance, 24% of yeast viability was encountered in the 30:70 EGO:CuNP combination at 500 μm/mL (*ca*. 29% for corresponding GO:CuNPs combination).

Although the yeast viability results shown in Figure 10c,d illustrated all antifungal activities were directly proportional to the increasing concentrations of the EGO and EGO-PEG in CuNPs combinations, the synergistic effects were not as encouraged as those observed in GO and GO-PEG. The best MIC_50_ values of 320 μm/mL and 265 μm/mL were measured only when very high ratio of CuNPs were employed in EGO:CuNPs and EGOPEG:CuNPs (Figure 10c,d). Nevertheless, the lowest viability value (15%) was indeed found when 99% of CuNPs was employed in EGO-PEG at the inhibitory concentration of 500 μg/mL (Table 1). This viability value was significantly lower than those were found in GO, GO-PEG and EGO (38%, 33% and 30%) with the same CuNPs ratio.

## 4. Conclusions

Coupling of PEG to the graphene-based nanomaterials, GO and EGO, via amide bond syntheses were achieved, these had subsequently yielded the two PEGylated products, GO-PEG and EGO-PEG (Figure 1). FTIR spectra of the resulting GO-PEG and EGO-PEG (Figure 2) confirmed the newly formed amide bonds (1732 and 1712 cm^−1^ NH-CO IR stretches) in both PEGylated products. Raman spectra of both PEGylated products GO-PEG and EGO-PEG (Figure 3b,d) confirmed that their carbonaceous structures remained unchanged after isolations from the coupling syntheses. Whilst SEM images showed the changes in the layered-structures in both PEGyalted GO and EGO, the corresponding TEM identified the directional and morphological changes of the lattice structures in both GO-PEG and EGO-PEG from their starting materials (Figure 4 and Figure 5). In addition, the changes in pH from the starting GO/EGO (pH 2.6–3.3) to give the neutralised GO-PEG and EGO-PEG products (pH 6.7–6.9) had further supported that the PEGylated had occurred (Figure 6a).

To assess the antifungal abilities and synergistic potentials of these graphene oxide derivatives, susceptibility tests of *C. albicans* were performed using the in vitro cut well method in the presence of different nanoparticle dispersants (0.1 *w*/*v*%). Among all the nanomaterials that were investigated (Figure 8a), CuNPs showed to be the most effective antifungal reagent against *C. albicans*, and the strength of antifungal efficacy was followed by GO-PEG > EGO-PEG > GO > EGO. These findings were in line with the MIC results that are shown in Figure 10. The viability results indicated that GO-PEG (Figure 10b) appeared to be as good antifungal agent as CuNPs, this can be explained by the high colloidal stability displayed in the corresponding zeta potential (−41 mV). The antifungal effect observed in GO-PEG was also supported by the SEM images (Figure 9), where physical interactions between GO-PEG and *C. albicans* had caused heavy damages and deformations of the spores and hyphae structures. It was also interesting to see the different morphological changes in *C. albicans* after treatments with different GO derivatives (Figure 9) and CuNPs (Figure 8e).

MIC studies suggested that the combination of graphene oxides and CuNPs at a ratio of 30:70 provided the optimal synergistic effects in the case of GO:CuNPs (Figure 10a) and GO-PEG:CuNPs (Figure 10b) where their MIC_50_ values were found to be between 185–225 μg/mL.

In summary, GO-PEG, GO:Cu (30:70), GO-PEG:Cu (30:70) and EGOPEG:CuNPs (1:99) showed to have good potential to be used as antifungal reagents against *Candida albicans*. From this study, we found that antifungal activities can be enhanced by manipulating the ratio of graphene oxide derivatives and CuNPs. This study supports the engineering of non-ingestible nanoparticles as potential components to be embedded or incorporated into biomedical dressings, instrument and/or other medical devices to combat fungal infections in the future.

## Figures and Tables

**Table 1:** *C. albicans* viability values obtained from IC 500 and 250 µg/mL of GO/EGO and PEGylated GO/EGO in different ratio of copper nanoparticles (CuNPs).

	Ratio of Different Graphene Oxide Derivatives to Copper Nanoparticles *							
	96:4		70:30		30:70		1:99	
Inhibitory Concentrations (µg/mL)	250	500	250	500	250	500	250	500
**GO**	90.6	86.0	73.8	58.8	31.6	28.5	69.5	38.1
**GO-PEG**	54.3	38.4	74.6	28.6	43.9	23.6	61.7	32.5
**EGO**	66.2	52.2	77.4	36.4	67.9	29.2	56.1	29.5
**EGO-PEG**	67.4	58.8	84.3	39.8	66.6	24.6	51.7	15.3

* 96:4 represents a combination of 96% of GO based material with 4% CuNPs; 70:30 represents a combination of 70% of GO based material with 30% CuNPs; 30:70 represent a combination of 30% of GO based material with 70% CuNPs; 1:99 represents a combination of 1% of GO based material with 99% CuNPs.

## References

[1] Robinson J.T., Perkins F.K., Snow E.S., Wei Z., Sheehan P.E. (2008). Reduced Graphene Oxide Molecular Sensors. Nano Lett..

[2] Huang C., Li C., Shi G. (2012). Graphene based catalysts. Energy Environ. Sci..

[3] Zhang S., Yang K., Feng L., Liu Z. (2011). In vitro and in vivo behaviors of dextran functionalized graphene. Carbon.

[4] Feng L., Liu Z. (2011). Graphene in biomedicine: Opportunities and challenges. Nanomedicine.

[5] Horváth L., Magrez A., Burghard M., Kern K., Forró L., Schwaller B. (2013). Evaluation of the toxicity of graphene derivatives on cells of the lung luminal surface. Carbon.

[6] Hong G., Diao S., Antaris A.L., Dai H. (2015). Carbon Nanomaterials for Biological Imaging and Nanomedicinal Therapy. Chem. Rev..

[7] Xu Z., Wang S., Li Y., Wang M., Shi P., Huang X. (2014). Covalent Functionalization of Graphene Oxide with Biocompatible Poly(ethylene glycol) for Delivery of Paclitaxel. ACS Appl. Mater. Interfaces.

[8] Raidongia K., Tan A.T.L., Huang J., Tanaka K., Iijima S. (2014). Chapter 14—Graphene Oxide: Some New Insights into an Old Material. Carbon Nanotubes and Graphene.

[9] Hirata M., Gotou T., Horiuchi S., Fujiwara M., Ohba M. (2004). Thin-film particles of graphite oxide 1. Carbon.

[10] Kovtyukhova N.I., Ollivier P.J., Martin B.R., Mallouk T.E., Chizhik S.A., Buzaneva E.V., Gorchinskiy A.D. (1999). Layer-by-Layer Assembly of Ultrathin Composite Films from Micron-Sized Graphite Oxide Sheets and Polycations. Chem. Mater..

[11] Lee S., Eom S.H., Chung J.S., Hur S.H. (2013). Large-scale production of high-quality reduced graphene oxide. Chem. Eng. J..

[12] Chen J., Peng H., Wang X., Shao F., Yuan Z., Han H. (2014). Graphene oxide exhibits broad-spectrum antimicrobial activity against bacterial phytopathogens and fungal conidia by intertwining and membrane perturbation. Nanoscale.

[13] Yadav N., Dubey A., Shukla S., Saini C.P., Gupta G., Priyadarshini R., Lochab B. (2017). Graphene Oxide-Coated Surface: Inhibition of Bacterial Biofilm Formation due to Specific Surface–Interface Interactions. ACS Omega.

[14] Sanchez V.C., Jachak A., Hurt R.H., Kane A.B. (2011). Biological Interactions of Graphene-Family Nanomaterials: An Interdisciplinary Review. Chem. Res. Toxicol..

[15] Zhang M., Li X.H., Gong Y.D., Zhao N.M., Zhang X.F. (2002). Properties and biocompatibility of chitosan films modified by blending with PEG. Biomaterials.

[16] Wu H., Liu G., Zhang S., Shi J., Zhang L., Chen Y., Chen F., Chen H. (2011). Biocompatibility, MR imaging and targeted drug delivery of a rattle-type magnetic mesoporous silica nanosphere system conjugated with PEG and cancer-cell-specific ligands. J. Mater. Chem..

[17] Prentice D.E., Majeed S.K. (1978). Oral toxicity of polyethylene glycol (PEG 200) in monkeys and rats. Toxicol. Lett..

[18] Leung H.-W., Ballantyne B., Hermansky S.J., Frantz S.W. (2016). Peroral Subchronic, Chronic Toxicity, and Pharmacokinetic Studies of a 100-KiIodaIton Polymer of Ethylene Oxide (Polyox N-10) in the Fischer 344 Rat. Int. J. Toxicol..

[19] Torchilin V.P., Omelyanenko V.G., Papisov M.I., Bogdanov A.A., Trubetskoy V.S., Herron J.N., Gentry C.A. (1994). Poly(ethylene glycol) on the liposome surface: On the mechanism of polymer-coated liposome longevity. Biochim. Biophys. Acta (Bba)—Biomembr..

[20] Zalipsky S. (1995). Chemistry of polyethylene glycol conjugates with biologically active molecules. Adv. Drug Deliv. Rev..

[21] Zhu S., Zhen H., Li Y., Wang P., Huang X., Shi P. (2014). PEGylated graphene oxide as a nanocarrier for podophyllotoxin. J. Nanoparticle Res..

[22] Shim G., Kim M.-G., Park J.Y., Oh Y.-K. (2016). Graphene-based nanosheets for delivery of chemotherapeutics and biological drugs. Adv. Drug Deliv. Rev..

[23] Shen J., Shi M., Li N., Yan B., Ma H., Hu Y., Ye M. (2010). Facile synthesis and application of Ag-chemically converted graphene nanocomposite. Nano Res..

[24] Ordikhani F., Ramezani Farani M., Dehghani M., Tamjid E., Simchi A. (2015). Physicochemical and biological properties of electrodeposited graphene oxide/chitosan films with drug-eluting capacity. Carbon.

[25] Mazaheri M., Akhavan O., Simchi A. (2014). Flexible bactericidal graphene oxide–chitosan layers for stem cell proliferation. Appl. Surf. Sci..

[26] Fan Z., Liu B., Wang J., Zhang S., Lin Q., Gong P., Ma L., Yang S. (2014). A Novel Wound Dressing Based on Ag/Graphene Polymer Hydrogel: Effectively Kill Bacteria and Accelerate Wound Healing. Adv. Funct. Mater..

[27] Habiba K., Bracho-Rincon D.P., Gonzalez-Feliciano J.A., Villalobos-Santos J.C., Makarov V.I., Ortiz D., Avalos J.A., Gonzalez C.I., Weiner B.R., Morell G. (2015). Synergistic antibacterial activity of PEGylated silver–graphene quantum dots nanocomposites. Appl. Mater. Today.

[28] Yapar N. (2014). Epidemiology and risk factors for invasive candidiasis. Ther. Clin. Risk Manag..

[29] Pfaller M.A., Diekema D.J. (2007). Epidemiology of Invasive Candidiasis: A Persistent Public Health Problem. Clin. Microbiol. Rev..

[30] Brown G.D., Denning D.W., Gow N.A.R., Levitz S.M., Netea M.G., White T.C. (2012). Hidden Killers: Human Fungal Infections. Sci. Transl. Med..

[31] Zaoutis T.E., Argon J., Chu J., Berlin J.A., Walsh T.J., Feudtner C. (2005). The Epidemiology and Attributable Outcomes of Candidemia in Adults and Children Hospitalized in the United States: A Propensity Analysis. Clin. Infect. Dis..

[32] Seddiki S.M.L., Boucherit-Otmani Z., Boucherit K., Kunkel D. (2015). Infectivités fongiques des cathéters implantés dues à Candida sp. Formation des biofilms et résistance. J. De Mycol. Médicale.

[33] Ramage G., Saville S.P., Thomas D.P., López-Ribot J.L. (2005). CandidaBiofilms: An Update: FIG. 1. Eukaryot. Cell.

[34] Taff H.T., Mitchell K.F., Edward J.A., Andes D.R. (2013). Mechanisms ofCandidabiofilm drug resistance. Future Microbiol..

[35] Maubon D., Garnaud C., Calandra T., Sanglard D., Cornet M. (2014). Resistance of Candida spp. to antifungal drugs in the ICU: Where are we now?. Intensive Care Med..

[36] Staniszewska M., Bondaryk M., Swoboda-Kopec E., Siennicka K., Sygitowicz G., Kurzatkowski W. (2013). Candida albicans morphologies revealed by scanning electron microscopy analysis. Braz. J. Microbiol..

[37] Ramachandran R., Chalasani A.G., Lal R., Roy U. (2014). A Broad-Spectrum Antimicrobial Activity of Bacillus subtilis RLID 12.1. Sci. World J..

[38] Pierce C.G., Uppuluri P., Tummala S., Lopez-Ribot J.L. (2010). A 96 Well Microtiter Plate-based Method for Monitoring Formation and Antifungal Susceptibility Testing of Candida Albicans Biofilms. J. Vis. Exp..

[39] Marcano D.C., Kosynkin D.V., Berlin J.M., Sinitskii A., Sun Z., Slesarev A., Alemany L.B., Lu W., Tour J.M. (2010). Improved Synthesis of Graphene Oxide. ACS Nano.

[40] Zheng H., Neo C.Y., Mei X., Qiu J., Ouyang J. (2012). Reduced graphene oxide films fabricated by gel coating and their application as platinum-free counter electrodes of highly efficient iodide/triiodide dye-sensitized solar cells. J. Mater. Chem..

[41] Wu J.-B., Lin M.-L., Cong X., Liu H.-N., Tan P.-H. (2018). Raman spectroscopy of graphene-based materials and its applications in related devices. Chem. Soc. Rev..

[42] Li M., Wang C. (2019). Preparation and characterization of GO/PEG photo-thermal conversion form-stable composite phase change materials. Renew. Energy.

[43] Lu J., Li Y., Li S., Jiang S.P. (2016). Self-assembled platinum nanoparticles on sulfonic acid-grafted graphene as effective electrocatalysts for methanol oxidation in direct methanol fuel cells. Sci. Rep..

[44] Mitchell A.P., Citiulo F., Jacobsen I.D., Miramón P., Schild L., Brunke S., Zipfel P., Brock M., Hube B., Wilson D. (2012). Candida albicans Scavenges Host Zinc via Pra1 during Endothelial Invasion. PLoS Pathog..

[45] Wilson D. (2019). Candida albicans. Trends Microbiol..

